# Supplementary material for: Inhibition of Insulin-like Growth Factor 1 Receptor/Insulin Receptor Signaling by Small-Molecule Inhibitor BMS-754807 Leads to Improved Survival in Experimental Esophageal Adenocarcinoma
Source: Cancers (Basel). 2024 Sep 17;16(18):3175. doi: 10.3390/cancers16183175 (PMC11430189; doi:10.3390/cancers16183175)
Supplement: Supplementary file 1 [file cancers-16-03175-s001.zip › cancers-3179881-supplementary.pdf]

Figure S1

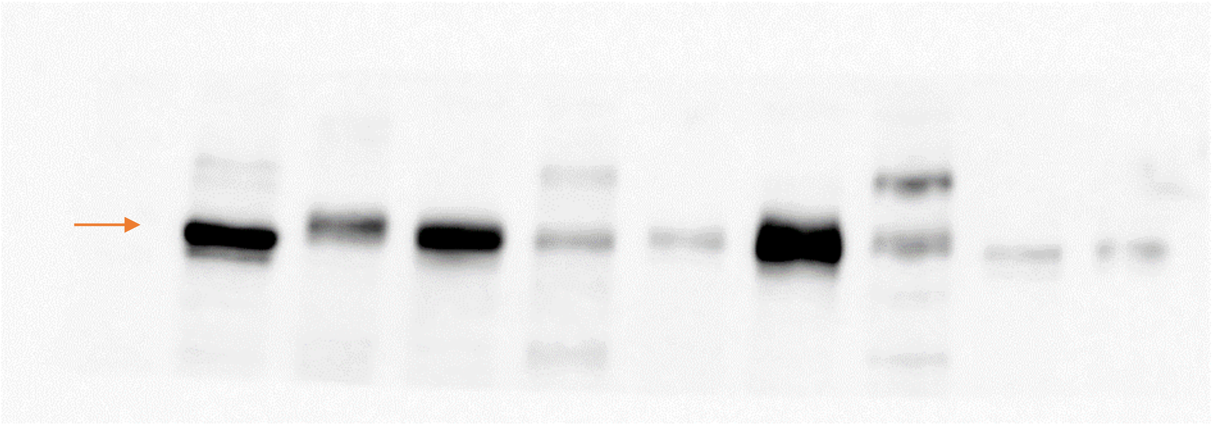

Phospho-IGF-1R/IR

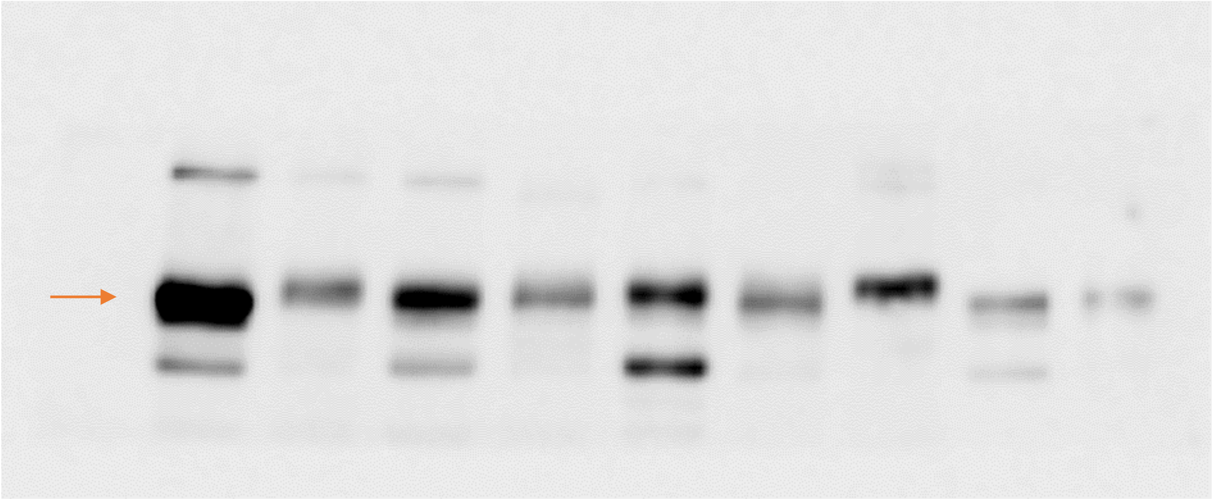

Total IGF-1R

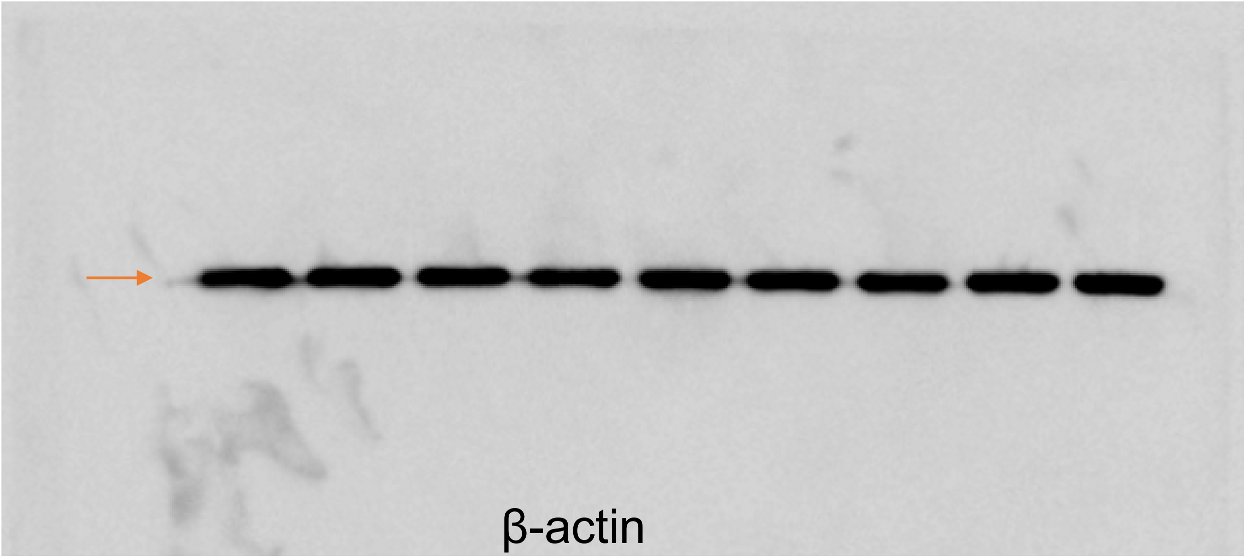

β-actin

Figure S3
(A)

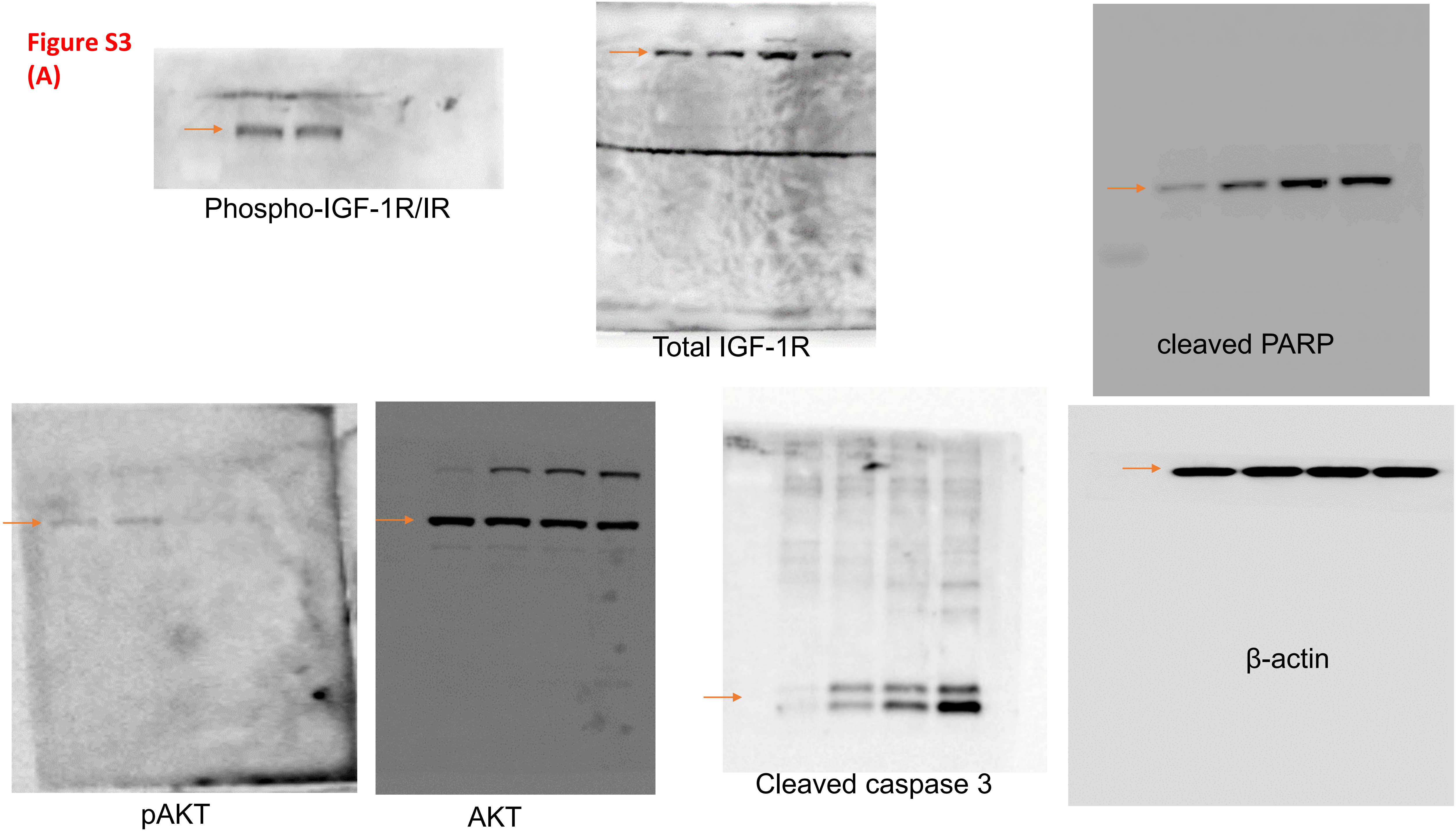
Phospho-IGF-1R/IR
Total IGF-1R
cleaved PARP
pAKT
AKT
Cleaved caspase 3
β-actin

Figure S3
(B)

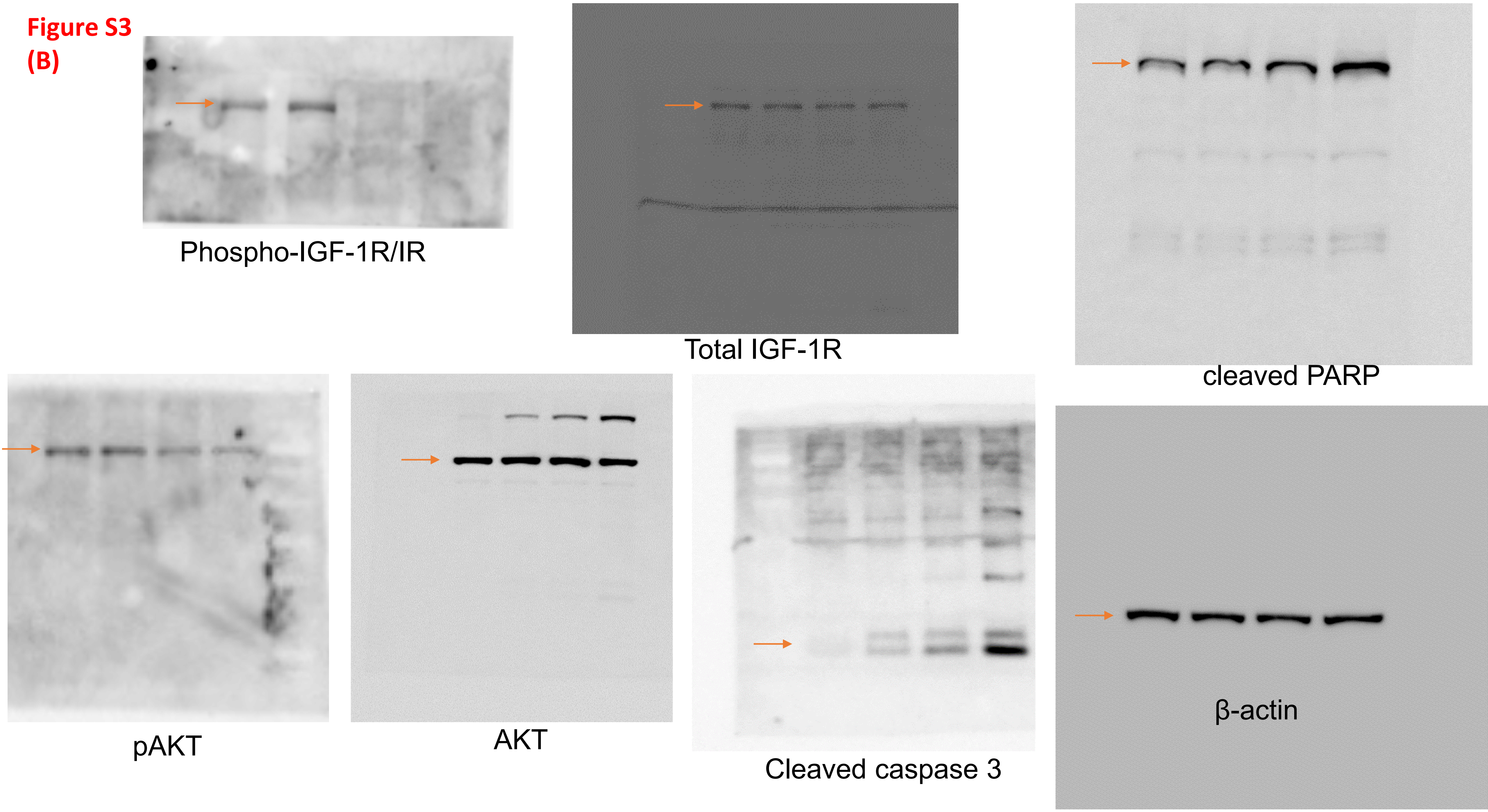
Phospho-IGF-1R/IR
Total IGF-1R
cleaved PARP
pAKT
AKT
Cleaved caspase 3
β-actin

Figure S3 (C)

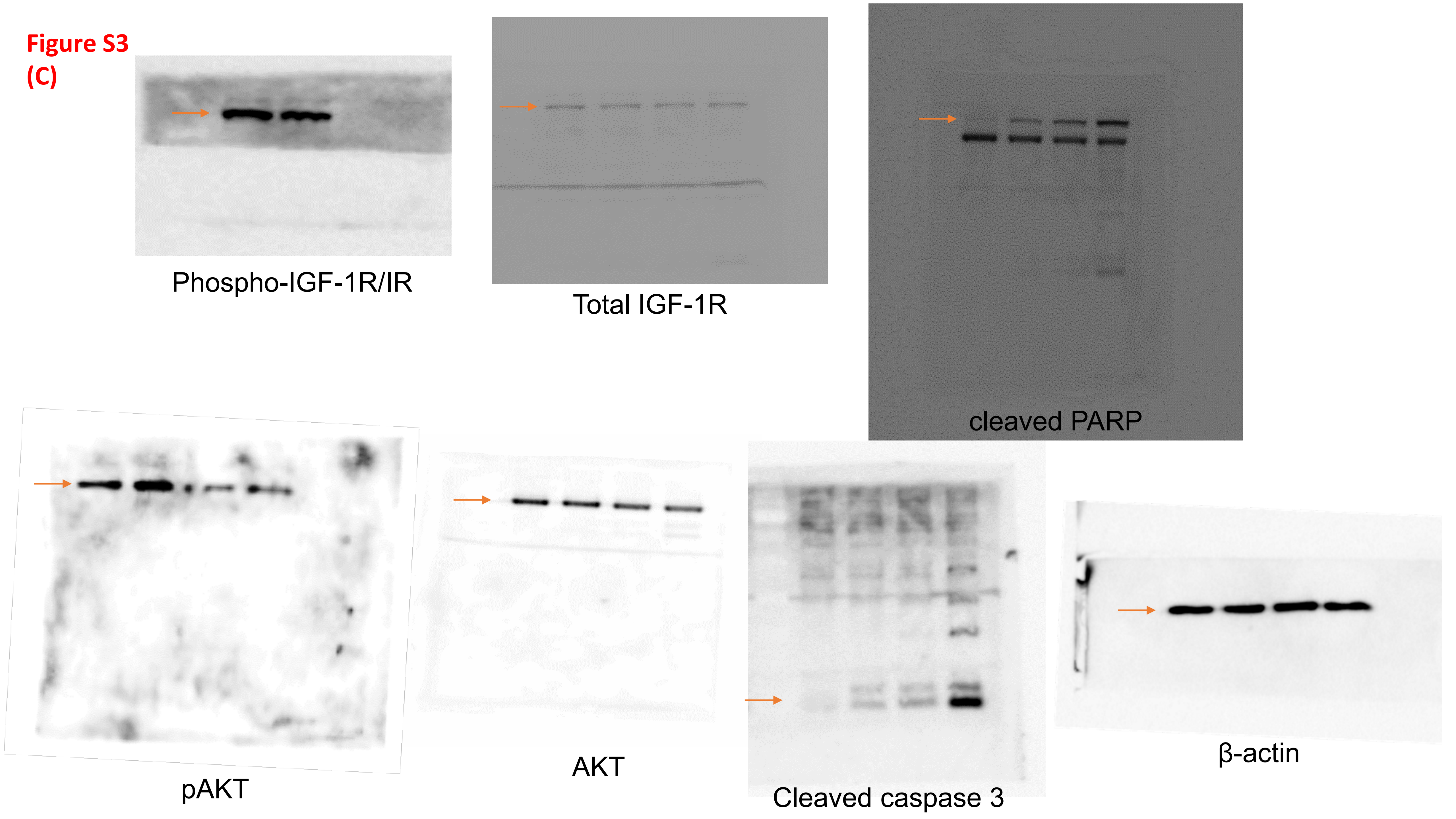
Phospho-IGF-1R/IR
Total IGF-1R
cleaved PARP
pAKT
AKT
Cleaved caspase 3
β-actin
